# Sorption Isotherms and Thermodynamic Characteristics of Gelatin Powder Extracted from Whitefish Skin: Mathematical Modeling Approach

**DOI:** 10.3390/foods13010092

**Published:** 2023-12-26

**Authors:** Mohammad Fikry, Soottawat Benjakul, Saleh Al-Ghamdi, Ajay Mittal, Krisana Nilsuwan, Ronnel Fulleros, Mokhtar Dabbour

**Affiliations:** 1 Department of Agricultural and Biosystems Engineering, Faculty of Agriculture, Benha University, Moshtohor, Toukh 13736, Egypt; mokhtar.dabbour@fagr.bu.edu.eg; 2 International Center of Excellence in Seafood Science and Innovation, Faculty of Agro-Industry, Prince of Songkla University, Hat Yai, Songkhla 90110, Thailand; ajy.mittal@yahoo.com (A.M.); krisana.n@psu.ac.th (K.N.); 3 Department of Agricultural Engineering, King Saud University, P.O. Box 2460, Riyadh 11451, Saudi Arabia; sasaleh@ksu.edu.sa (S.A.-G.); rfulleros@ksu.edu.sa (R.F.)

**Keywords:** fish gelatin, dynamic sorption isotherms, isosteric heat of sorption, differential entropy, enthalpy

## Abstract

Moisture adsorption and desorption isotherms of gelatin extracted from whitefish skin powder (FSGP) at different temperatures across a wide range of water activity were determined along with their thermodynamic properties. Nine mathematical models were utilized for fitting the experimental data and simulating the adsorption and desorption behavior. The thermodynamic properties were determined and fitted to the experimental data. The results showed that Peleg and GAB models were the best fit for FSGP. The energies involved in the adsorption and desorption process of FSGP indicated a stronger dependence on equilibrium moisture content (X_e_). When X_e_ decreased, there was a consistent trend of increasing thermodynamic properties. Both the moisture adsorption and desorption behaviors of FSGP were, therefore, non-spontaneous processes. Linear correlations between the changes in enthalpy and entropy for adsorption and desorption were observed, indicating the presence of enthalpy–entropy compensation for FSGP. For preserving FSGP quality, it should be stored with X_w_ ≤ 8 (g_w_/g_dm_, d.b.) at temperatures below 53 °C and an RH of 50% to avoid it becoming rubbery. These findings are crucial for providing insight into the optimal drying and storage conditions.

## 1. Introduction

With a growing awareness of Halal compliance among Muslims, their concerns extend beyond meat-based products to encompass a broader range of items such as food, cosmetics, personal care goods, and medications. Technological advancements have resulted in the availability of various products that may contain either permissible (Halal) or prohibited (Haram) components. Gelatin is thermally denatured collagen from bones, hides, organs, and skins from animals like pigs, fish, cows, chickens, and others [1]. The global demand for gelatin has been steadily increasing, reaching an annual production output of 677,200 tons in 2021 [2]. The predominant sources of gelatin are pork and beef skin or bones. Within the total gelatin usage, pig skin gelatin accounts for the largest share at 46%, followed by bovine hides (29.4%), bones (23.1%), and other sources (1.5%) [3]. Fish gelatin production constitutes a minor portion, contributing only about 1% to the world’s annual gelatin production. Gelatin finds widespread uses as a food ingredient, serving as a gelling and foaming agent, as well as in the formulation of pharmaceutical products like soft and hard capsules and microspheres. It also plays a role in wound dressing and three-dimensional tissue regeneration in the biomedical field, along with various non-food applications like photography [4,5].

Gelatin powder is widely recognized for its hygroscopic nature due to its propensity to absorb moisture from the surrounding environment. As a consequence, its stability is reduced during extended storage [6]. This moisture absorption/desorption causes noticeable changes in the powder’s macroscopic properties, such as stickiness, caking, and shrinkage [7]. These changes adversely affect the powder’s quality, making it difficult to handle and impeding its flow. The magnitude of these changes is influenced by the temperature differential between the ambient temperature and the gelatin powder’s glass transition temperature. The glass transition temperature refers to the point at which a solid “glass” system undergoes a transition into a rubbery state with liquid-like characteristics [8]. A larger temperature difference results in a faster rate of macroscopic property changes [9]. To maintain the stability of gelatin powder during storage, it is advisable to store it below its glass transition temperature (37 °C) or critical water activity (0.2–0.4), below which the chemical reactions are insignificant during storage [7,10].

For describing the behavior of adsorption and desorption of the food powder, moisture sorption isotherms represent the relationship between a food’s equilibrium moisture content (X_e_) and its corresponding water activity (a_w_) across a range of a_w_ values while maintaining a constant temperature [11]. Valuable insights into the water binding strength of a solid material can be elucidated [12]. These isotherms serve as a crucial tool for estimating the water retention capacity of a material when exposed to specific temperature and relative humidity conditions.

Various methods are available for determining moisture sorption isotherms, in which the gravimetric method is commonly used [10,11,13,14,15]. This method involves measuring weight changes, which can be performed continuously or discontinuously in dynamic or static systems. Continuous methods utilize electro-balances or quartz spring balances to measure weight changes, while discontinuous systems involve placing salt or sulfuric acid solutions in vacuum or atmospheric conditions together with the food material to assess the equilibrium relative humidity (ERH) [16]. The disadvantages associated with discontinuous systems include slow equilibrium processes and potential mold or bacterial growth at high relative humidity (RH). However, such technical difficulties can be overcome by employing the dynamic vapor sorption (DVS) method. Unlike the use of saturated salt solutions, the DVS system utilizes a mixture of dry nitrogen and saturated water vapor to achieve the desired RH. This setup facilitates faster equilibration due to the small sample chamber and continuous flow of dry nitrogen. Additionally, the DVS system eliminates the risk of microorganism growth at high RH, allowing for measurements of absorption and desorption isotherms using the same sample [11,17].

Investigating the moisture sorption isotherms offers significant thermodynamic properties that hold significant relevance in the design and sizing of equipment for various preservation processes [18,19]. These properties encompass enthalpy, entropy, Gibbs free energy, and the enthalpy–entropy compensation theory. Those aforementioned parameters play a critical role in elucidating the reactions and phenomena taking place at the molecular level within food materials [20].

On the other hand, mathematical modeling plays a vital role in determining the sorption isotherms and their thermodynamic characteristics. Several mathematical models have been suggested to characterize the sorption properties of moisture in food such as Oswin [21], modified Oswin [22], Peleg [23], GAB [24], Halsey [25], modified Halsey [26], BET [27,28] modified Henderson [29], and Adam and Shove [30].

Therefore, extensive studies on this topic have previously been conducted by various researchers, including Rahman, et al. [6], Aguirre-Álvarez, et al. [10], Fikry and Al-Awaadh [11], Hassan, et al. [15], Antoniolli, et al. [18], Sormoli and Langrish [20], Rahman and Al-Belushi [31], Argyropoulos, et al. [32], and Stępień, et al. [33]. However, to the best of the authors’ knowledge, no published works on moisture sorption of whitefish skin gelatin powder (FSGP) under various temperatures and its thermodynamic characteristics are available. Hence, this work was conducted to investigate the desorption isotherms of FSGP across a broad spectrum of relative humidity (RH) levels at different temperatures, to identify the most appropriate model for fitting the adsorption and desorption isotherms of FSGP, to estimate the thermodynamic characteristics associated with the adsorption and desorption of moisture in FSGP, and to explore how varying moisture content affects the glass transition temperature (T_g_) of FSGP, specifically examining its plasticizing effect.

## 2. Materials and Methods

### 2.1. Sorption Isotherm Determination Using Dynamic Vapor Sorption (DVS) System

**Figure 1:**
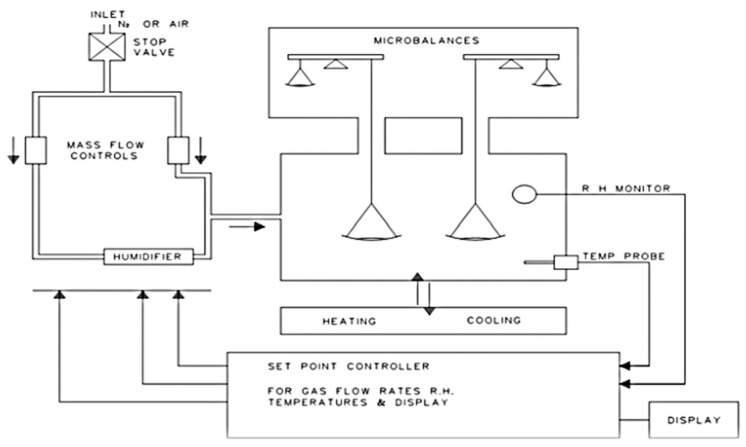
Systematic diagram of AQUADYNE DVS.

Food-grade gelatin with a 250 bloom value, sourced from whitefish skin, was procured from the Vinh Hoan Company, located in Cao Lanh City, Dong Thap Province, Vietnam. The fish gelatin samples’ sorption isotherms were assessed employing an AQUADVS dynamic vapor sorption (DVS) instrument (Quantachrome Instruments, Boynton Beach, FL, USA). This equipment is composed of multiple elements, including an electronic microbalance, a humidifier, electronic mass flow controllers, a relative humidity monitor, a temperature regulator, and a microprocessor linked to a computer. Figure 1 illustrates the instrument configuration.

Adhering to the approach detailed by Fikry and Al-Awaadh [11], pre-dehydrated FSGP samples (100 ± 5 mg), with an initial moisture content (X_w_) of 0.08 ± 0.01 (g_w_/g_dm_ d.b.), were employed for the diverse experiments. The experiments involved ascertaining sorption isotherms at distinct temperatures (25, 35, and 45 °C) while upholding a constant temperature. The range of water activities spanned from 0.09 to 0.83. To ensure precision and dependability, each test was performed in duplicates. At each step of relative humidity, the equilibrium moisture content (X_e_) of the sample, quantified as kilograms of water per kilogram of dry solids, was computed to construct the sorption isotherms.

### 2.2. Modeling Sorption Isotherms Data

Nine widely used mathematical models were employed to fit the sorption data. Table 1 comprises the proposed mathematical models, applied to the experimental adsorption and desorption data of FSGP.

### 2.3. Spreading Pressure

The determination of the surface excess free energy, termed the spreading pressure (∅), was computed utilizing the method outlined by Iglesias, et al. [34]:

(1) $$\emptyset =\frac{K_{B}T}{A_{m}}\int_{0}^{a_{w}}\frac{\theta }{a_{w}}da_{w}$$

where *θ* refers to the moisture ratio, which can be calculated as follows: $\theta =X/X_{m}$. $K_{B}$ represents Boltzmann’s constant (1.38 × 10^−23^ J/K), T stands for temperature in Kelvin, and *A_m_* denotes the area of a water molecule (1.06 × 10^−19^ m^2^). The observed spreading pressure was found to be moderate at *a_w_* = 0. The process of computing the integral in Equation (1) shifted from a numerical method to adopting an empirical correlation between water activity and moisture content. Consequently, the Dent model [35] (Equation (2)) was employed in this study.

(2) $$\frac{a_{w}}{X}=\frac{1}{b_{o}X_{m}}+\frac{b_{o}-2b}{b_{o}X_{m}}a_{w}-\frac{b(b_{o}-b)}{b_{o}X_{m}}a_{w}^{2}$$

Here, *b* and *b_o_* stand for constants derived from the Dent sorption isotherm, reflecting characteristics of the adsorbed water (dimensionless). Through nonlinear regression analysis, the values of *b* and *b_o_* in Equation (2) were determined. This was accomplished by utilizing the monolayer moisture content, computed via the BET equation in Table 1, along with experimental data on equilibrium moisture relationships. By substituting Equation (2) into Equation (1), reorganizing the terms, and subsequently integrating, the spreading pressure was derived based on the surface area per sorption site or the area occupied by a single water molecule on each sorption site.

(3) $$\emptyset =\frac{K_{B}T}{A_{m}}ln\left[\frac{1+b_{o}a_{w}-ba_{w}}{1-ba_{w}}\right]$$

Equation (3) was applied to ascertain the spreading pressure values under various temperatures for both adsorption and desorption processes.

### 2.4. Thermodynamic Characteristics of FSGP

The heat of adsorption signifies the energy liberated during sorption, while the heat of desorption pertains to the energy necessitated to disrupt the intermolecular bonds between water vapor molecules and the adsorbent’s surface [36]. The heat of sorption serves as an indicator of the attractive forces between the sorption sites and water vapor molecules [37]. As such, the interplay between moisture content and water activity holds paramount importance in delineating this energy relationship.

The net isosteric heat of sorption (*q_st_*) or the differential enthalpy of sorption (ΔH) can be deduced from the analysis of moisture sorption data using Equation (4). In this specific investigation, the Clausius–Clapeyron equation, expounded in Equation (5) by Gomez, et al. [38], was employed for the computation of *q_st_*. For the present study, the isosteric heat of sorption and the differential entropy (Δ*S*) of water adsorption and desorption at varying levels of moisture content were acquired by fitting Equation (6) to the equilibrium moisture content data. Through the graphing of ln(*a_w_*) against 1/T at a consistent moisture content, the isosteric heat of sorption was ascertained based on the gradient, akin to −*q_st_*/*R*. The Δ*S* value was derived using the coefficient of linearity (Δ*S*/*R*). This procedure was iterated for each equilibrium moisture content to delve into the connection between *q_st_* and the equilibrium moisture content. It is important to emphasize that this technique assumes a consistent isosteric heat of sorption across diverse temperatures, and this methodology necessitates sorption data being collected at a minimum of three different temperatures [14].

(4) $$q_{st}=Q_{st}-\lambda$$

(5) $$\left[\frac{d\ln\left(a_{w}\right)}{d(\frac{1}{T})}\right]=-\frac{q_{st}}{R}$$

(6) $$\ln\left(a_{w}\right)=-\frac{q_{st}}{RT}+\frac{∆S}{R}$$

Equation (7) can be employed to compute the vaporization heat (*λ*) of pure water (J/mol). While Equation (8) was used to determine the Gibbs free energy (∆G).

(7) $$\lambda =R\left[6687-5.31*T\right]$$

(8) $$∆G=-RT\ln\left(a_{w}\right)$$

The connection between the enthalpies (Δ*H*) and entropies (Δ*S*) in a set of interconnected reactions, as expressed in Equation (9), is referred to as the isokinetic relationship. The parameter denoted as $T_{\beta }$, known as the isokinetic temperature (relationship slope), signifies a temperature at which all reactions within the series are anticipated to occur at the same rate [39].

(9) $$∆H={∆G}_{\beta }+T_{\beta }∆S$$

The harmonic mean temperature (*T_h_*) can be determined using Equation (10).

(10) $$T_{h}=\frac{n}{\sum_{i=1}^{n}\left(1/T\right)}$$

where *Q_st_* signifies the isosteric heat of sorption; *R* stands for the universal gas constant (8.314 J/mol·K); *T*, $T_{\beta }$, and $T_{h}$ indicate the absolute, isokinetic, and harmonic mean temperatures (K), respectively; Δ*S* denotes the differential entropy (J/mol·K); Δ*H* stands for the differential enthalpy or net isosteric sorption heat, *q_st_*, (J/mol); Δ*G* and ${∆G}_{\beta }$ represent the Gibbs free energy and Gibbs free energy at $T_{\beta }$ (kJ/mol), respectively; and *n* refers to the number of isotherms.

### 2.5. Measurement of Glass Transition Temperature

According to the procedure used by Rahman, et al. [6], the FSGP samples were exposed to saturated salt solutions at room temperature for one week to facilitate moisture adsorption and then the determination of the gelatin samples’ glass transition temperature at various moisture contents was carried out using a DSC instrument (DSC Ql0, TA Instruments, New Castle, DE, USA). A mechanical refrigerated cooling system was utilized to cool the sample to −80 °C. The calibration of the instrument for heat flow and temperature was conducted using distilled water (melting point = 0 °C; ∆H_m_ = 334 J/g) and indium (melting point = 156.5 °C; ∆H_m_ = 28.5 J/g). Aluminum pans with a capacity of 30 μL, equipped with lids for sealing, were used in all experiments. An empty sealed pan served as the reference. Nitrogen gas was used as the carrier gas, flowing at a rate of 50 mL/min.

For each experiment, 6 ± 1 mg of gelatin sample was placed in an aluminum pan and sealed. The sealed pan with the sample was then cooled to −80 °C at a rate of 5 °C/min and equilibrated for 10 min. Subsequently, the sample was scanned from −80 °C to 300 °C at an optimum heating rate 10 °C/min (with or without 30 min of annealing at *T_g_* ± 1 °C). Each thermogram obtained was analyzed to determine the onset, mid-, and end points of the glass transition. The characteristic temperature of the glass transition was determined as the midpoint of the transition.

### 2.6. Water Plasticization Behavior

The model proposed by Gordon and Taylor [40] (Equation (11)) was employed to fit the glass transition temperature and moisture content data in order to estimate the plasticization effect of water.

(11) $$T_{g}=\frac{\left(1-X_{w}\right)T_{gs}+kX_{w}T_{gw}}{\left(1-X_{w}\right)+kX_{w}}$$

The model includes the glass transition temperatures of the mixture (*T_g_*), solids (*T_gs_*), and water (*T_gw_* = −135 °C) [41], with *X_w_* representing the mass fraction of water and *k* denoting the model constant.

### 2.7. Data Analysis

To evaluate the effectiveness of the proposed mathematical models in fitting the experimental data (Equations (12)–(14)), three statistical measures were employed: the coefficient of determination (R^2^), the mean relative percent error (E %), and the sum of squared errors (SSEs). Calculation of these statistical parameters was performed using the Minitab18 software. The selection of the optimal model was determined based on the following criteria: achieving the highest correlation coefficient R^2^ (≥0.90), the lowest PE % (below 10%), and the minimum SSE values (close to zero), as emphasized by [11,42].

(12) $$R^{2}=\frac{\sum_{i=1}^{N}{\left(X_{pred}-\bar{X_{exp}}\right)}^{2}}{\sum_{i=1}^{N}{\left(X_{exp}-\bar{X_{exp}}\right)}^{2}}$$

(13) $$SSE=\frac{1}{N}\sum_{i=1}^{N}{{(X}_{exp}-X_{pred})}^{2}$$

(14) $$E\left(\%\right)=\frac{100}{N}\;\sum_{i=1}^{N}\frac{\left|X_{exp}-X_{pred}\right|}{X_{exp}}$$

In the context of the equation, $X_{exp}$, $X_{pred}$, and $\bar{X_{exp}}$ stand for the experimental, predicted, and average moisture content values (g_w_/g_dm_ d.b.), respectively. N represents the total number of experimental data points.

## 3. Results and Discussion

### 3.1. Profile of Moisture Sorption of FSGP

**Figure 2:**
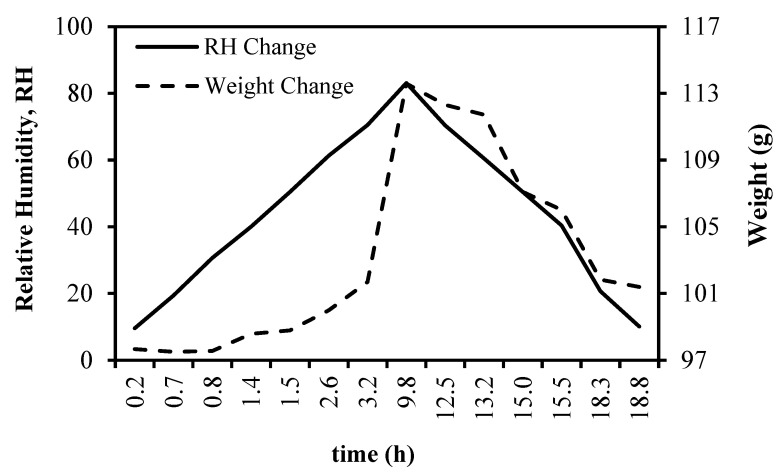
Time-based representation of moisture sorption for FSGP subjected to gradual shifts in equilibrium relative humidity (ERH) and sample weight at 25 °C.

An illustrative instance of the time-dependent pattern of moisture sorption for FSGP at 25 °C, utilizing the DVS system, is depicted in Figure 2. The graph illustrates that the sample’s weight increased when the relative humidity rose during the adsorption phase, while the reverse occurred during desorption. The state of equilibrium was achieved within 10 h for each process.

### 3.2. Adsorption and Desorption Isotherms of FSGP

**Figure 3:**
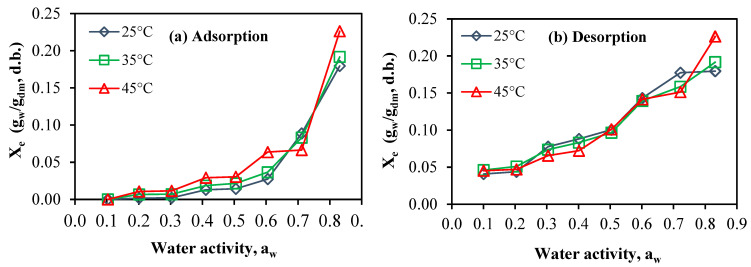
The curves of sorption isotherms: (**a**) for adsorption and (**b**) for desorption, acquired for FSGP across temperatures of 25, 35, and 45 °C.

Figure 3 portrays the experimental outcomes of adsorption and desorption isotherms acquired at various temperatures for FSGP. The adsorption isotherms aligned with a type III configuration, as classified by the BET criterion [43]. This result signifies that FSGP had a minimum moisture content at low relative humidity and greater amounts at higher relative humidity. These observations are in harmony with those of date powder rich in fiber [11] and lemon peel [44].

The upsurge in the equilibrium moisture content (X_e_) was evident with increasing a_w_ at a consistent temperature during the adsorption phase (Figure 3a), while X_e_ diminished with declining a_w_ throughout the desorption process (Figure 3b). A parallel pattern was identified in analogous products such as date powder rich in fiber [11], citrus reticulata leaves [45], leaves and stems of lemon balm (Argyropoulos et al., 2012), Tunisian olive leaves (Bahloul et al., 2008) and orange peel and leaves [14,32,45,46].

Remarkably, the temperature had no discernible impact on the sorption isotherms of FSGP, indicating that it likely did not induce a significant elevation in the water molecules’ excitation state [47]. Similar results were also documented for date fiber [11] and safflower petals and tarragon [48].

### 3.3. Moisture Sorption Hysteresis

Moisture sorption hysteresis is a phenomenon characterized by the existence of two distinct paths between adsorption and desorption isotherms. The degree of hysteresis is linked to the composition and state of components within a food product. It can reflect changes in their structure and conformation, which in turn affect the accessibility of polar sites with favorable energy characteristics, potentially impeding moisture movement [49].

**Figure 4:**
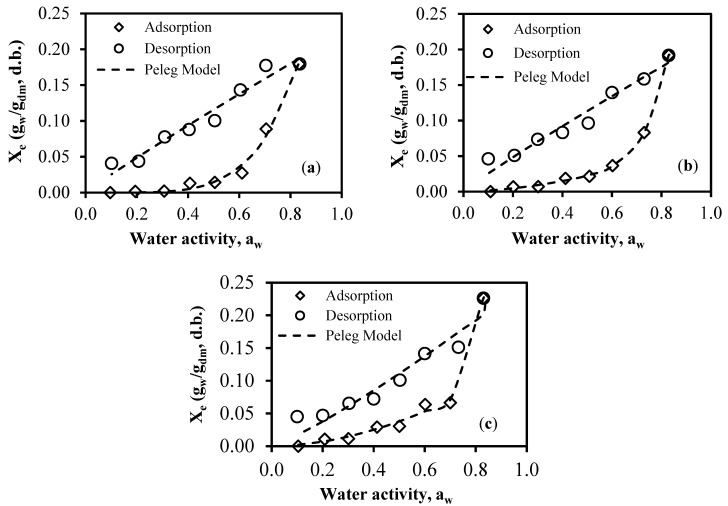
Observed and predicted sorption data (desorption and adsorption) for the FSGP obtained at (**a**) 25 °C, (**b**) 35 °C, and (**c**) 45 °C (hysteresis loop).

Hysteresis loops were observed at temperatures of 25 °C, 35 °C, and 45 °C, as depicted in Figure 4. This phenomenon could be explained by the concept of a solid structure pore connected to its surroundings via a small capillary. According to this explanation, during adsorption, the capillary begins to be filled due to rising relative humidity, when the pore remains empty. In contrast, during desorption, the pore is initially saturated with liquid. The liquid can only escape when the air’s vapor pressure becomes lower than the liquid’s vapor pressure inside the capillary. Because the system of pores typically encompasses a wide range of capillary diameters, variations between adsorption and desorption behaviors are observed [45]. It was noted that at identical water activity, X_e_ was greater for the desorption curve compared to the adsorption curve, as illustrated in Figure 4 at temperatures of 25 °C, 35 °C, and 45 °C. This hysteresis phenomenon may be attributed to the pre-drying that the samples underwent during the adsorption process. Similar behavior has been observed by [10] Fikry and Al-Awaadh [11], and Kammoun Bejar, et al. [46].

### 3.4. Modeling the Sorption Data of FSGP

Table 2 provides an overview of the estimated parameters for the sorption models used for describing the moisture adsorption and desorption of FSGP, while Table 3 shows the fitting criteria of the proposed models. Specifically, the Peleg model (no. 1) proved to be suitable for accurately representing the experimental adsorption isotherm data at different temperatures (25, 35, and 45 °C) across a broad spectrum of water activity (0.09–0.83). While, the GAB model was the best model to adequately describe the desorption isotherm of FSGP. Figure 3 visually shows the projected sorption isotherms based on the most suitable model (Peleg model). A similar result has been found for fish gelatin from different sources [10].

### 3.5. Spreading Pressure of FSGP

**Figure 5:**
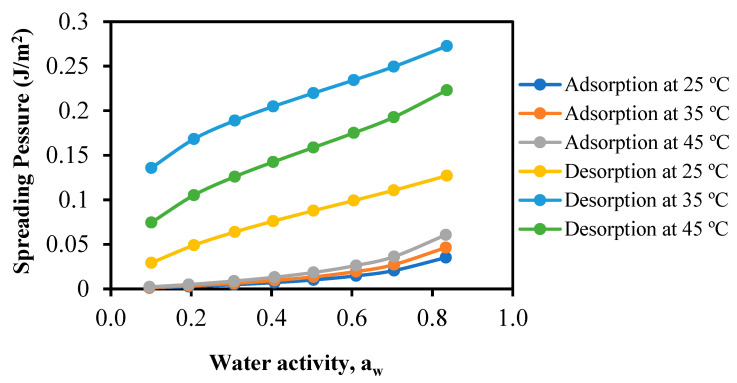
Spreading pressures of FSGP at various water activities and temperatures.

The values of the constants b and b_0_ in Equation (2) at temperatures of 25, 35, and 45 °C were determined as follows: 0.0536, 0.0720, and 0.0655, respectively, for adsorption (with R^2^ values of 0.985, 0.992, and 0.982). For desorption, the values were 0.0392, 0.0397, and 0.0418, respectively, with R^2^ values of 0.901, 0.925, and 0.968. The spreading pressures of FSGP are displayed in Figure 5. These findings indicate that the spreading pressures of the desorption process were greater than those for the adsorption process. Moreover, the spreading pressure increased as the water activity rose at any given temperature. These outcomes could be explained by the hysteresis phenomenon, which reflects the changes in their structure and conformation, which in turn affect the accessibility of polar sites with favorable energy characteristics, potentially impeding moisture movement [48]. A similar trend has been found for walnut kernels [50].

### 3.6. Thermodynamic Properties of FSGP

Thermodynamic characteristics hold a significant role in the formulation of drying process designs [11]. The thermodynamic properties, such as the differential heat of sorption, stand as fundamental indicators of the intricate interactions between whitefish skin gelatin powder (FSGP) and moisture. This property reveals the energy changes during moisture sorption, depicting the varying energy requirements as FSGP binds water molecules at different moisture levels [49]. These properties provide pivotal insights into FSGP’s stability and performance across diverse applications. They aid in designing optimal storage conditions and processes, ensuring product integrity, and contribute to the development of accurate models for predicting FSGP behavior under differing environmental settings.

The values associated with the different heats of sorption are crucial for energy computations [49]. Alterations in the Q_st_ represent a quantification of the energy fluctuations that transpire when water molecules combine with the sorbent during sorption operations [51]. Entropy reflects the level of disorder present within the water–sorbent system and facilitates the comprehension of processes like dissolution, crystallization, and swelling [51].

**Figure 6:**
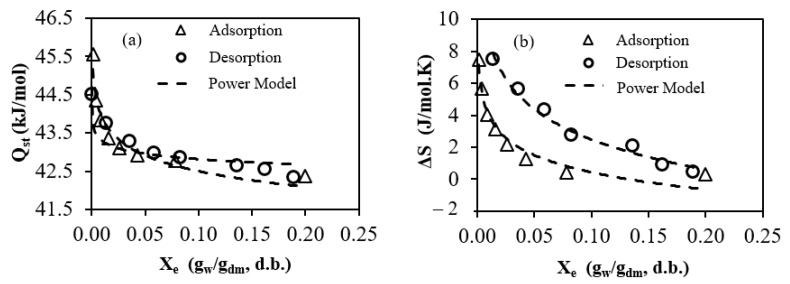
Isosteric heat of sorption (**a**) and differential entropy (**b**) plotted against the equilibrium moisture content of FSGP.

Figure 6a presents the correlation between moisture content and the overall isosteric heats of adsorption and desorption for FSGP. The findings reveal an inverse relationship, in which the equilibrium moisture content decreases, whereas the isosteric heat increases. Evidently, the heat of sorption is notably elevated at low moisture content levels, suggesting the presence of robust water molecule bonds with the food matrix, forming a monolayer of molecules. Consequently, detaching these water molecules demands substantial energy expenditure [52]. These outcomes align with those observed in certain food materials investigated previously, including olive leaves [14], date fiber [11], and tea [53]. It has been documented that the heat of sorption acts as an indicator of the intermolecular attractive forces between the sorption sites and water vapor [54]. The relationship between Q_st_ and X_e_ is effectively modeled using power functions, as detailed below:

(15) $${\left(Q_{st}\right)}_{Ad}=41.16X_{e}^{-0.014}{\;\;\;\;\;\;\;\;R}^{2}=0.948$$

(16) $${\left(Q_{st}\right)}_{De}=42.36X_{e}^{-0.005}{\;\;\;\;\;\;\;\;R}^{2}=0.844$$

Figure 6b displays the connection between differential entropy and equilibrium moisture content for both the adsorption and desorption isotherms of FSGP. The outcomes reveal a strong correlation between the entropy data and equilibrium moisture content. Notably, the adsorption phase displays lower values of deffrential entropy compared to desorption, suggesting that water molecules exhibit greater mobility during desorption than during adsorption [16]. Similar findings were formerly observed for starch powder [16] and tow mints [55].

The deffrential entropy (∆*S*) is linked to the binding forces within the system and is related to spatial arrangements at the water–sorbent interface. These results were consistent with the concept that a lower deffrential entropy corresponds to restricted molecular movement when the product has high water content. The correlation between the deffrential entropy and moisture content can be predicted using the logarithmic functions

(17) $${\left(∆S\right)}_{Ad}=-3.19\ln X_{e}-4.76{\;\;\;\;\;\;R}^{2}=0.988$$

(18) $${\left(∆S\right)}_{De}=-1.54\ln X_{e}-3.12{\;\;\;\;\;\;R}^{2}=0.963$$

From a thermodynamic perspective, the Gibbs free energy (ΔG) can serve as an indicator of the affinity between water and the sorbent material. Additionally, it can offer insight whether the sorption of water is a spontaneous or non-spontaneous process, which depends on the sign (whether it is negative or positive) of the ΔG values [56].

**Figure 7:**
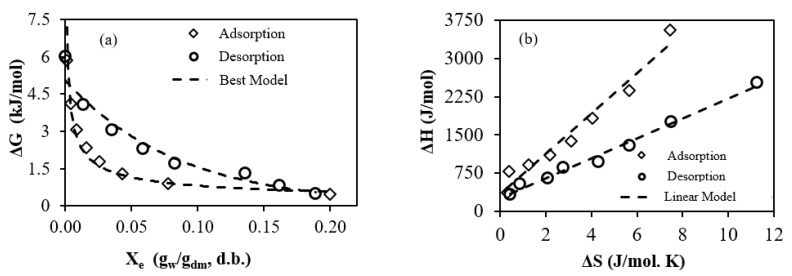
Gibbs free energy (ΔG) plotted against X_e_ of FSGP (**a**) and enthalpy–entropy compensation of FSGP (**b**).

The ΔG values observed for FSGP (Figure 7a) indicated that both moisture adsorption and desorption behaviors were non-spontaneous processes, as they exhibited positive ΔG values. This behavior aligned with the typical expectations for sorption processes, which require the input of energy to occur, making them endergonic reactions [15,56]. Similar non-spontaneous processes have also been previously observed [15,56].

Additionally, the results demonstrated that ΔG increased as the equilibrium moisture content (X_e_) decreased. This phenomenon of rising ΔG with decreasing X_e_ was also reported for jambolan [56].

Furthermore, the changes in adsorption and desorption ΔG followed a power and exponential model for FSGP, respectively. The lines depicted in Figure 7a,b represent the fits of these respective models to the ΔG data. These models yielded excellent fits (R^2^ > 0.97), as can be seen in Equations (19) and (20).

(19) $${\left(∆G\right)}_{Ad}=0.26X_{e}^{-0.5}{\;\;\;\;\;\;\;\;R}^{2}=0.974$$

(20) $${\left(∆G\right)}_{De}=5.04e^{{-11.8X}_{e}}{\;\;\;\;\;\;\;\;R}^{2}=0.971$$

To investigate the connection between the physical and chemical events potentially occurring during the moisture desorption process of FSGP (as shown in Figure 7b), the enthalpy–entropy compensation theory was examined. To minimize alterations in the free energy of these events, compensation arises due to changes in the ΔH or ΔS values, which are influenced by the interaction between the solvent and solute. Consequently, the relationship between ΔH and ΔS exhibits a linear pattern for a particular reaction [56].

A linear correlation between the changes in enthalpy (ΔH) and entropy (ΔS) was observed, as illustrated in Figure 7b. This observation indicated the presence of enthalpy–entropy compensation for FSGP. By applying Equation (9), the isokinetic temperatures (T_β_) for adsorption and desorption were 393.9 and 195.75 K (Equations (21) and (22)), respectively, in which all reactions of adsorption and desorption occurred at the same rate. The intercept of Equation (9) corresponds to the free energy change (ΔG_β_), reflecting the affinity of the sorbent for water. Furthermore, a positive ΔG_β_ suggests a non-spontaneous process [56]. The harmonic mean temperature (T_h_) calculated from Equation (10) was 307.8 K. Since T_β_ was bigger than T_h_, the moisture adsorption process was considered as being enthalpy-driven.

(21) $${\left(∆H\right)}_{Ad}=393.9∆S+336.92{\;\;\;\;\;\;\;\;R}^{2}=0.962$$

(22) $${\left(∆H\right)}_{De}=195.75∆S+260.18{\;\;\;\;\;\;\;\;R}^{2}=0.988$$

### 3.7. Glass Transition Temperature and Water Plasticization Behavior

**Figure 8:**
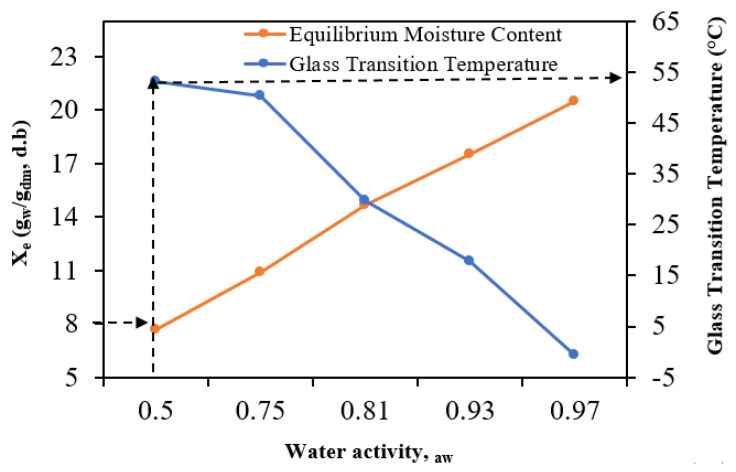
Glass transition temperature (T_g_) of FSGP versus water activity and moisture content (X_e_). Dotted arrows indicate the glass transition temperature, below which the FSGP should be stored at a moisture content of (8 g_w_/g_dm_, d.b.) and a relative humidity of 50%.

In Figure 8, the T_g_ values for various samples are plotted against their moisture contents and water activities. Notably, there is a noticeable effect of water plasticization, as an increase in moisture content leads to a reduction in the glass transition temperatures. This phenomenon is consistent with findings in other food products such as tomato pulp, papaya, and freeze-dried pineapple [57,58,59,60].

Figure 8 also illustrates that the actual data can be effectively projected using the Gordon and Taylor model (Equation (11)). This model, known for its reliability, has consistently demonstrated its ability to forecast the glass transition temperatures of FSGP at varying moisture levels. It was fitted to the experimental data points using a T_gw_ (glass transition temperature) value of −135 °C, and nonlinear regression yielded the following parameters: T_gs_ (starting temperature) = 3.4 °C and k (slope) = 0.33, with an R^2^ value of 0.934. Based on the insights from Figure 8, it is advisable to store FSGP with moisture content (X_w_) less than or equal to 8 (g_w_/g_dm_, d.b.) at temperatures below 53 °C and RH of 50% to prevent it from transitioning into a rubbery state, which could lead to issues like agglomeration and stickiness.

## 4. Conclusions

The moisture sorption isotherms for FSGP were determined using a DVS apparatus at three different temperatures (25 °C, 35 °C, and 45 °C) and over a wide range of water activity (0.09–0.83). The results revealed that FSGP exhibited type III sorption behavior. As the relative humidity increased, moisture adsorption also increased, while the opposite trend was observed during desorption. Notably, the hysteresis phenomenon was evident.

The equilibrium moisture data of FSGP were effectively fitted to the Peleg model for adsorption process, while the desorption data were adequately described by GAB model. The findings also indicated that the spreading pressures of desorption process were greater than those for adsorption process. Moreover, the spreading pressure increased as the water activity rose at any given temperature. Additionally, both the isosteric heats of sorption and the differential entropy of FSGP exhibited a decline as the moisture content increased, suggesting a significant dependency on moisture content. The observed ΔG values indicated that both moisture adsorption and desorption processes for FSGP were non-spontaneous. Linear correlations were found between changes in enthalpy (ΔH) and entropy (ΔS) for both adsorption and desorption, suggesting the presence of enthalpy–entropy compensation in FSGP. To prevent FSGP from turning rubbery, store it with X_w_ ≤ 8 (g_w_/g_dm_, d.b.) at temperatures below 53 °C and RH of 50%. The importance of these results lies in their contribution to a better understanding of moisture sorption behavior, modeling, and storage conditions for FSGP, which is valuable for maintaining product quality and preventing undesirable texture changes in practical applications.

These findings contribute significantly to understanding FSGP’s moisture sorption behavior, aiding in the development of accurate models and optimal storage conditions. This knowledge is crucial for maintaining FSGP quality across industries like food, pharmaceuticals, and biomedicine.

Future research in whitefish skin gelatin powder (FSGP) could explore several avenues. Advanced modeling techniques integrating additional variables like temperature fluctuations or additives could refine predictive models, aiding precise predictions for diverse applications. Investigating the impact of processing methods such as spray drying or freeze-drying on FSGP’s moisture sorption behavior would optimize production and quality. Long-term stability studies and functional applications across industries like pharmaceuticals or biomedicine could provide insights into FSGP’s shelf life and varied uses. Moreover, exploring innovative storage conditions and preservation techniques, assessing environmental impacts, and investigating potential bioactive properties could further broaden FSGP’s applications and enhance its overall understanding and utility.

## Figures and Tables

**Table 1:** Mathematical models applied to the experimental sorption isotherm data of FSGP.

No	Model Name	Equation	Reference
1	Peleg	$X_{e}=C_{1}{\left(a_{w}\right)}^{C_{2}}+C_{3}{\left(a_{w}\right)}^{C_{4}}$	[23]
2	GAB	$X_{e}=\frac{X_{m\;}CK\;a_{w}}{\left(1-Ka_{w}\right)\left(1-Ka_{w}+CK\;a_{w}\right)}$	[24]
3	BET	$X_{e}=\frac{X_{m\;}Ca_{w}}{\left[\left(1-a_{w}\right)+\left(C-1)(1-a_{w}\right)a_{w}\right]}$	[27]
4	Halsey	$X_{e}={\left(-\frac{A}{\ln a_{w}}\right)}^{1/B}$	[25]
5	Oswin	$X_{e}=K{\left(a_{w}/1-a_{w}\right)}^{n}$	[21]
6	Smith	$X_{e}=A-B\left(ln\left(1-a_{w}\right)\right)$	[28]
7	Adam and Shove	$X_{e}=A+Ba_{w}+C{a_{w}}^{2}+D{a_{w}}^{3}$	[30]
8	Modified Oswin	$X_{e}=\left(A+BT\right){\left[\frac{a_{w}}{\left(1-a_{w}\right)}\right]}^{C}$	[22]
9	Modified Halsey	$X_{e}={\left[-\frac{exp\left(A+BT\right)}{lna_{w}}\right]}^{C}$	[26]

Note: X_e_ is the equilibrium moisture content (g/g dry solid); X_m_ is the monolayer moisture content (g/g dry solid); A, B, C, C_1_, C_2_, C_3_, C_4_, D, K, and n are model constants (dimensionless).

**Table 2:** The estimated parameters of the models utilized for the experimental sorption data of FSGP at varying temperatures.

Parameter	Adsorption			Desorption		
	25 °C	35 °C	45 °C	25 °C	35 °C	45 °C
Peleg model $X_{e}=C_{1}{\left(a_{w}\right)}^{C_{2}}+C_{3}{\left(a_{w}\right)}^{C_{4}}$						
C_1_	0.43	0.83	191.97	0.22	0.21	0.25
C_2_	4.91	9.40	39.18	0.94	0.93	1.17
C_3_	0.02	0.07	0.14	0.00	0.00	0.00
C_4_	4.91	1.65	1.84	8.74	3.85	5.13
GAB model $X_{e}=\frac{X_{m\;}CK\;a_{w}}{\left(1-Ka_{w}\right)\left(1-Ka_{w}+CK\;a_{w}\right)}$						
X_m_	1.84	0.03	0.02	0.71	0.07	0.06
C	0.01	0.49	2.73	3.08	10.32	13.83
K	0.91	1.06	1.11	0.11	0.77	0.89
BET model $X_{e}=\frac{X_{m\;}ca_{w}}{\left[\left(1-a_{w}\right)+\left(c-1)(1-a_{w}\right)a_{w}\right]}$						
X_m_	0.05	0.07	0.07	0.04	0.04	0.04
C	0.26	0.17	0.28	5.50 × 10^7^	1.25 × 10^8^	7.10 × 10^7^
Halsey model $X_{e}={\left(-\frac{A}{\ln a_{w}}\right)}^{1/B}$						
A	0.06	0.07	0.07	0.01	0.01	0.02
B	0.64	0.61	0.66	1.97	1.84	1.54
Oswin model $X_{e}=k{\left(a_{w}/1-a_{w}\right)}^{n}$						
k	0.02	0.02	0.03	0.11	0.10	0.10
n	1.31	1.39	1.27	0.39	0.41	0.49
Smith model $X_{e}=A-B\left(ln\left(1-a_{w}\right)\right)$						
A	−0.04	−0.03	−0.03	0.04	0.04	0.03
B	0.10	0.11	0.12	0.09	0.09	0.11
Adam and Shove model $X_{e}=A+Ba_{w}+C{a_{w}}^{2}+D{a_{w}}^{3}$						
A	−0.02	−0.05	−0.06	0.05	0.04	0.04
B	0.28	0.58	0.70	−0.18	0.00	0.07
C	−1.00	−1.73	−2.01	0.93	0.30	0.00
D	1.14	1.66	1.88	−0.63	-0.11	0.21
Modified Oswin model $X_{e}=\left(A+BT\right){\left[\frac{a_{w}}{\left(1-a_{w}\right)}\right]}^{C}$						
A	6.64	0.13	14.64	−4.24	0.42	−0.78
B	−0.26	0.00	−0.33	0.17	−0.01	0.02
C	1.31	1.30	1.23	0.39	0.41	0.49
Modified Halsey model $X_{e}={\left[-\frac{exp\left(A+BT\right)}{lna_{w}}\right]}^{C}$						
A	122.81	−3.70	312.23	−1.93	−23.84	−275.21
B	−5.02	0.03	−7.06	−0.12	0.55	6.08
C	1.56	1.68	1.24	0.51	0.53	0.71

**Table 3:** The fitting criteria of the models utilized for the experimental sorption data of FSGP at varying temperatures.

Parameter	Adsorption			Desorption		
	25 °C	35 °C	45 °C	25 °C	35 °C	45 °C
Peleg model $X_{e}=C_{1}{\left(a_{w}\right)}^{C_{2}}+C_{3}{\left(a_{w}\right)}^{C_{4}}$						
SSE	3.64 × 10^−5^	3.03 × 10^−6^	2.68 × 10^−5^	1.25 × 10^−4^	1.20 × 10^−4^	2.88 × 10^−4^
R^2^	0.990	0.999	0.994	0.953	0.951	0.917
E (%)	0.16	0.03	0.11	0.15	0.17	0.34
GAB model $X_{e}=\frac{X_{m\;}CK\;a_{w}}{\left(1-Ka_{w}\right)\left(1-Ka_{w}+CK\;a_{w}\right)}$						
X_m_	1.84	0.03	0.02	0.71	0.07	0.06
SSE	7.44 × 10^−5^	4.23 × 10^−6^	5.30 × 10^−5^	1.28 × 10^−4^	6.04 × 10^−5^	1.08 × 10^−4^
R^2^	0.979	0.999	0.989	0.952	0.975	0.969
E (%)	0.42	0.03	0.11	0.16	0.07	0.10
BET model $X_{e}=\frac{X_{m\;}ca_{w}}{\left[\left(1-a_{w}\right)+\left(c-1)(1-a_{w}\right)a_{w}\right]}$						
SSE	8.01 × 10^−5^	5.56 × 10^−6^	9.70 × 10^−5^	1.12 × 10^−3^	5.31 × 10^−4^	2.67 × 10^−4^
R^2^	0.977	0.998	0.979	0.576	0.782	0.923
E (%)	0.67	0.14	0.22	0.52	0.29	0.17
Halsey model $X_{e}={\left(-\frac{A}{\ln a_{w}}\right)}^{1/B}$						
SSE	1.03 × 10^−4^	4.40 × 10^−6^	7.68 × 10^−5^	3.71 × 10^−4^	1.06 × 10^−4^	1.07 × 10^−4^
R^2^	0.970	0.999	0.984	0.860	0.957	0.969
E (%)	0.71	0.03	0.13	0.35	0.10	0.10
Oswin model $X_{e}=k{\left(a_{w}/1-a_{w}\right)}^{n}$						
SSE	8.14 × 10^−5^	6.43 × 10^−6^	9.49 × 10^−5^	2.52 × 10^−4^	6.41 × 10^−5^	1.03 × 10^−4^
R^2^	0.977	0.998	0.980	0.905	0.974	0.970
E (%)	0.41	0.05	0.20	0.20	0.06	0.11
Smith model $X_{e}=A-B\left(ln\left(1-a_{w}\right)\right)$						
SSE	4.36 × 10^−4^	4.82 × 10^−4^	6.78 × 10^−4^	2.62 × 10^−4^	6.05 × 10^−5^	9.91 × 10^−5^
R^2^	0.875	0.868	0.856	0.901	0.975	0.971
E (%)	2.74	1.19	1.05	0.18	0.05	0.08
Adam and Shove model $X_{e}=A+Ba_{w}+C{a_{w}}^{2}+D{a_{w}}^{3}$						
SSE	3.27 × 10^−5^	4.67 × 10^−5^	1.77 × 10^−4^	6.95 × 10^−5^	3.51 × 10^−5^	1.07 × 10^−4^
R^2^	0.991	0.987	0.962	0.974	0.986	0.969
E (%)	0.22	0.20	0.41	0.07	0.03	0.08
Modified Oswin model $X_{e}=\left(A+BT\right){\left[\frac{a_{w}}{\left(1-a_{w}\right)}\right]}^{C}$						
SSE	8.14 × 10^−5^	6.55 × 10^−5^	1.41 × 10^−1^	2.52 × 10^−4^	7.15 × 10^−5^	2.03 × 10^−4^
R^2^	0.977	0.999	0.982	0.905	0.974	0.970
E (%)	0.41	0.07	100.89	0.20	0.07	0.18
Modified Halsey model $X_{e}={\left[-\frac{exp\left(A+BT\right)}{lna_{w}}\right]}^{C}$						
SSE	1.03 × 10^−4^	1.46 × 10^−5^	7.00 × 10^−3^	5.37 × 10^−3^	1.98 × 10^−4^	1.57 × 10^−1^
R^2^	0.970	0.999	0.990	0.860	0.961	0.980
E (%)	0.71	0.07	38.42	4.93	0.17	76.51

## Data Availability

Data is contained within the article.
